# The association between pre-miR-27a rs895819 polymorphism and myocardial infarction risk in a Chinese Han population

**DOI:** 10.1186/s12944-017-0652-x

**Published:** 2018-01-06

**Authors:** Meng-yun Cai, Jie Cheng, Meng-yuan Zhou, Li-li Liang, Si-min Lian, Xiao-shan Xie, Shun Xu, Xinguang Liu, Xing-dong Xiong

**Affiliations:** 1 Institute of Aging Research, Guangdong Medical University, Xin Cheng Avenue 1#, Songshan Lake, Dongguan, 523808 People’s Republic of China; 2 Guangdong Provincial Key Laboratory of Medical Molecular Diagnostics, Guangdong Medical University, Dongguan, People’s Republic of China; 3 Department of Clinical Laboratory, The Affiliated Hospital of Guangdong Medical University, Zhanjiang, China; 4 Institute of Biochemistry & Molecular Biology, Guangdong Medical University, Zhanjiang, People’s Republic of China

**Keywords:** miR-27a, Single nucleotide polymorphism, rs895819, Myocardial infarction, Disease susceptibility

## Abstract

**Background:**

Accumulating evidences have shown that miRNAs are directly or indirectly involved in a variety of biological processes, and closely associated with diverse human diseases, including cardiovascular diseases. SNPs locating within pri/pre-miRNA can affect miRNA processing and binding ability of target genes. MiR-27a, miR-26a-1 miR-100, miR-126 and miR-218 were reported to be associated with pathogenesis of myocardial infarction (MI). Here we aimed to evaluate the potential association of five polymorphisms in these pri/pre-miRNAs with individual susceptibility to MI in a Chinese Han population.

**Methods:**

Genotyping was performed in 287 MI cases and 646 control subjects using polymerase chain reaction-ligase detection reaction (PCR-LDR) method. The association of these SNPs with MI risk was performed with SPSS software.

**Results:**

In a logistic regression analysis, we found that AG heterozygote (OR = 0.40, 95% CI = 0.21-0.76, *P*a = 0.005) or AA homozygote (OR = 0.40, 95% CI = 0.22-0.75, *P*a = 0.004) of pre-miR-27a rs895819 had a reduced susceptibility to MI in comparison with GG homozygote. Similarly, a reduced risk of MI was detected when the AG and AA genotypes were combined (OR = 0.40, 95% CI = 0.22-0.74, *P*a = 0.003). However, no significant association between pri-miR-26a-1 pri-miR-100, pri-miR-126 and pri-miR-218 polymorphisms and MI risk was observed under the allelic and established genetic models. Further stratified analysis of pre-miR-27a rs895819 revealed a more significant association of AG + AA genotypes with MI risk among younger, male and smoking subjects. Interestingly, AG and AA genotypes of the rs895819 polymorphism conferred about 0.17 mmol/L and 0.18 mmol/L increase in HDL-C levels compared to GG genotype.

**Conclusions:**

Our findings suggest that the pre-miR-27a rs895819 polymorphism is associated with MI susceptibility in the Chinese Han population, which probably due to influence the HDL-C levels.

**Electronic supplementary material:**

The online version of this article (10.1186/s12944-017-0652-x) contains supplementary material, which is available to authorized users.

## Background

Myocardial infarction (MI) is a leading cause of death and disability, which has posed major challenges for China’s health system [1–3]. Previously established risk factors for MI including hypertension, diabetes, physical inactivity, smoking, alcohol intake, abnormal lipids, abdominal obesity, high-risk diet and psychosocial stress factors [4]. However, these modifiable factors can not readily explain overall MI incidence, thus hereditary factors must be involved. It is believed that complex interaction between susceptibility genes and environmental risk factors contributes to the occurrence of coronary artery disease (CAD) and its extreme manifestation of MI, and single nucleotide polymorphisms (SNPs), as the most common genetic variation was much accounted for [5, 6].

MicroRNAs (miRNAs) are a class of small non-coding RNA molecules with a length of 18~25 nucleotides. Typically, miRNAs function as the suppressors by guiding the miRNA-induced silencing complex (miRISC) to the 3′ untranslated regions (3’UTRs) of mRNA targets. Accumulating evidences have shown that miRNAs are directly or indirectly involved in a variety of biological processes, and closely associated with diverse human diseases, including cancer [7], Alzheimer’s disease [8] and cardiovascular diseases [9, 10]. MiRNA biogenesis begins with transcription by RNA polymerase II to create the primary miRNA (pri-miRNA). Then miRNA precursor (pre-miRNA) is generated from pri-miRNA by the nuclear ribonuclease (RNase) III DROSHA and the RNA-binding protein DCGR8. Ultimately, the precursor is exported out of the nucleus and processed the precursor into the mature miRNA by a RNase III enzyme, Dicer [11]. Biogenesis of miRNA could be regulated at each of these steps. Previous studies have demonstrated that SNP locating within pri-miRNAs or pre-miRNAs can affect miRNA processing and binding ability of target genes [12].

According to previous studies, miR-27a, miR-26a-1 miR-100, miR-126 and miR-218 were reported to be associated with pathogenesis of MI [13–17]. In this study, we conducted a large-scale case-control association study in 287 MI patients and 646 controls to investigate the correlation between five polymorphisms in these pri/pre-miRNAs and MI susceptibility, and revealed that the pre-miR-27a rs895819 polymorphism is associated with MI susceptibility in the Chinese Han population, which probably due to influence the HDL-C levels.

## Methods

### Study population

In this case-control study, 287 MI patients and 646 control subjects were consecutively recruited from the First People’s Hospital of Foshan (Foshan, China) and the Affiliated Hospital of Guangdong Medical University (Zhanjiang, China) from March 2011 to October 2015. We consulted each subject for the genetic relatedness information and excluded the individuals related to the subjects who had enrolled in this study. Therefore, all subjects were genetically unrelated Han Chinese, as describe before [18]. The diagnosis of MI was based on unstable angina and typical electrocardiographic changes such as significant ST-segment-T wave (ST-T) changes, new left bundle branch block, development of pathological Q waves, also on increases in the serum cardiac markers, such as creatinine kinase, aspartate aminotransferase, lactate dehydrogenase and troponin T. The diagnosis was further confirmed by the identification of the responsible stenosis in any of the major coronary arteries or in the left main trunk by coronary angiography. All the MI patients were newly diagnosed and previously untreated. A total of 646 controls were judged to be free of MI by questionnaires, medical history, clinical examination and electrocardiography. Besides, we excluded subjects with chronic ischemic heart disease from the control group by electrocardiogram, treadmill exercise test and coronary computed tomography angiography. The diagnosis of hypertension was established if patients were on antihypertensive medication or if the mean of 3 measurements of systolic blood pressure (SBP) above 140 mmHg or diastolic blood pressure (DBP) above 90 mmHg, respectively. Diabetes mellitus was defined as fasting blood glucose (FBG) above 7.0 mmol/L or use anti-diabetic drug therapies. Individuals that smoked once a day for over 1 year were defined as smokers. Hyperlipidemia was defined as serum total cholesterol (TC) concentration > 5.72 mmol/L or triglyceride (TG) concentration > 1.70 mmol/L or use of lipid-lowering therapy. In addition, individuals with medication treatment (statins, fibrates, diuretics, beta-blockers and hormones), congestive heart failure, peripheral vascular disease, rheumatic heart disease, pulmonary heart disease, chronic kidney, hepatic disease or any malignancy were excluded by questionnaires and medical history from this study. Information on demographic data and MI-related risk factors was collected by a structured questionnaire after obtaining the informed consent. This study was approved by the Medical Ethics Committee of the First People’s Hospital of Foshan and the Affiliated Hospital of Guangdong Medical University.

### Biochemical parameters analysis

After an informed consent, 2 ml of peripheral blood sample was collected from each subject for later DNA extraction and genotyping assays. All of the participants of MI and control groups were required to blood taken in the morning and after fasted for at least 8 h. The levels of plasma total cholesterol (TC), triglyceride (TG), high-density lipoprotein cholesterol (HDL-C) and low-density lipoprotein cholesterol (LDL-C) were measured enzymatically using a chemistry analyzer (Olympus, Japan). Glucose was analyzed by the glucose oxidase method with an Abbott V/P Analyzer (Abbott Laboratories, USA).

### DNA extraction

Genomic DNA was isolated from peripheral whole blood using blood DNA extraction kit (TianGen Biotech, Beijing, China) according to the manufacturer’s instructions. All DNA samples were dissolved in water and stored at −20 °C until use.

### Genotyping

SNP genotyping were performed utilizing polymerase chain reaction-ligase detection reaction (PCR-LDR) method (Shanghai Biowing Applied Biotechnology Company), as described in our previous study [18]. The sequence of primers and probes were showed in Additional file 1: Table S1.

### Statistical analysis

Hardy-Weinberg equilibrium was tested using the goodness-of-fit χ^2^ test among the control subjects. Qualitative variables were expressed as percentages and quantitative variables were expressed as mean ± standard deviation (SD). The differences of the demographic characteristics between the cases and controls were estimated using the χ^2^ test or Student’s *t* test for categorical variables and continuous variables respectively. Association between rs895819 and the risk of MI was evaluated using logistic regression analysis. The odds ratios (ORs) and 95% confidence intervals (CIs) for the effect of rs895819 on MI risk adjusted by age, sex, smoking, drinking, hypertension, diabetes and hyperlipidemia. One-way analysis of variance (ANOVA) was performed to analyze the association between SNP and lipid profiles. All the analyses were performed using the SPSS software (version 21), and *P* value of less than 0.05 was used as the criterion of statistical significance.

## Results

### Association of pri/pre-miRNA polymorphisms with the risk of MI

Two hundred eighty seven MI cases and 646 control subjects were included in the current study. Basic characteristics of the study objects were presented in Table 1. Compared with control subjects, the MI cases had higher rate of male, smoking and alcohol consumers, prevalence of hypertension, hyperlipidemia and diabetes, and higher levels of fasting plasma glucose (FBG), Triglycerides (TG), and LDL-C, but lower HDL-C. These dataset demonstrated that the male gender, alcohol intake, smoking, hypertension, diabetes and dyslipidemia were the important and well-known risk factors for developing MI in this study.

**Table 1:** The characteristics of MI cases and controls

Variable	Cases (*n* = 287)	Controls (*n* = 646)	*P*-value ^**a**^
Age (years)	61.82 ± 11.98	61.44 ± 12.28	0.660
Sex (male)	222 (77.4%)	374 (57.9%)	**<0.001** ^b^
Smoking	171 (59.6%)	164 (25.4%)	**<0.001**
Drinking	77 (26.8%)	91(14.1%)	**<0.001**
Hypertension	182 (63.4%)	227 (35.1%)	**<0.001**
Diabetes	137 (47.7%)	105 (16.3%)	**<0.001**
Hyperlipidemia	203 (70.7%)	238 (36.8%)	**<0.001**
Systolic BP (mm Hg)	140.18 ± 19.18	132.20 ± 18.73	**<0.001**
Diastolic BP (mm Hg)	75.67 ± 11.53	72.73 ± 10.39	**<0.001**
FPG (mmol/L)	6.63 ± 1.73	5.80 ± 1.91	**<0.001**
Triglycerides (mmol/L)	2.05 ± 0.96	1.48 ± 0.80	**<0.001**
Total cholesterol (mmol/L)	4.71 ± 1.22	4.62 ± 1.14	0.305
LDL cholesterol (mmol/L)	3.04 ± 0.97	2.63 ± 0.92	**<0.001**
HDL cholesterol (mmol/L)	1.19 ± 0.39	1.38 ± 0.67	**<0.001**

^a^Two-sided chi-square test or independent-samples *t*-test

^b^ *P* values under 0.05 were indicated in bold font

The observed allele and genotype distributions of these pri/pre-miRNA polymorphisms in the cases and controls were listed in Table 2. The observed genotype distribution in the controls did not deviate from the Hardy–Weinberg equilibrium (*P* > 0.05), providing no evidence of population stratification within the dataset.

**Table 2:** Multivariate associations of the pre-miR-27a rs895819 with the risk of MI

Model	Genotype	Cases (n = 287)	Controls (n = 646)	OR (95% CI) ^a^	*P* ^a^
		No. (%)	No. (%)		
*pre-miR-27a rs895819 HWE* ^b^ *: 0.4179*					
Allele	G	160 (27.9)	325 (25.2)	1.00	
	A	414 (72.1)	967 (74.8)	0.77 (0.60-1.01)	0.055
Genotype	GG	29 (10.1)	37 (5.7)	1.00	
	AG	102 (35.5)	251 (38.9)	0.40 (0.21-0.76)	0.005
	AA	156 (54.4)	358 (55.4)	0.40 (0.22-0.75)	0.004
Dominant	AG + GG	288 (44.6)	131 (45.6)	1.00	
	AA	156 (54.4)	358 (55.4)	0.86 (0.62-1.21)	0.389
Recessive	GG	29 (10.1)	37 (5.7)	1.00	
	AG + AA	258 (89.9)	609 (94.3)	0.40 (0.22-0.74)	0.003
*pri-miR-26a-1 rs7372209 HWE: 0.7195*					
Allele	C	433 (75.4)	980 (75.9)	1.00	
	T	141 (24.6)	312 (24.1)	0.98 (0.74-1.29)	0.879
Genotype	TT	16 (5.6)	36 (5.6)	1.00	
	CT	109 (38.0)	240 (37.2)	1.26 (0.58-2.76)	0.556
	CC	162 (56.4)	370 (57.3)	1.21 (0.57-2.60)	0.619
Dominant	CC	162 (56.4)	370 (57.3)	1.00	
	CT + TT	125 (43.6)	276 (42.7)	1.01 (0.72-1.42)	0.947
Recessive	TT	16 (5.6)	36 (5.6)	1.00	
	CT + CC	271 (94.4)	610 (94.4)	1.23 (0.58-2.61)	0.585
*pri-miR-100 rs1834306 HWE: 0.2017*					
Allele	C	341 (59.4)	742 (57.4)	1.00	
	T	233 (40.6)	550 (42.6)	0.97 (0.77-1.23)	0.816
Genotype	TT	45 (15.7)	125 (19.3)	1.00	
	CT	143 (49.8)	300 (46.4)	1.28 (0.80-2.05)	0.303
	CC	99 (34.5)	221 (34.2)	1.13 (0.69-1.85)	0.628
Dominant	TT	45 (15.7)	125 (19.3)	1.00	
	CT + CC	242 (84.3)	521 (80.7)	1.22 (0.78-1.90)	0.391
Recessive	CC	99 (34.5)	221 (34.2)	1.00	
	CT + TT	188 (65.5)	425 (65.8)	1.06 (0.75-1.51)	0.733
*pri-miR-126 rs4636297 HWE: 0.4457*					
Allele	G	497 (86.6)	1106 (85.6)	1.00	
	A	77 (13.4)	186 (14.4)	0.95 (0.68-1.33)	0.755
Genotype	GG	216 (75.3)	471 (72.9)	1.00	
	AG	65 (22.6)	164 (25.4)	1.01 (0.69-1.48)	0.951
	AA	6 (2.1)	11 (1.7)	1.44 (0.41-5.10)	0.569
Dominant	AG + GG	281 (97.9)	635 (98.3)	1.00	
	AA	6 (2.1)	11 (1.7)	1.44 (0.41-5.07)	0.571
Recessive	GG	216 (75.3)	471 (72.9)	1.00	
	AG + AA	71 (24.7)	175 (27.1)	1.04 (0.71-1.50)	0.857
*pri-miR-218 rs11134527 HWE: 0.4264*					
Allele	A	331 (57.7)	726 (56.2)	1.00	
	G	243 (42.3)	566 (43.8)	1.08 (0.84-1.38)	0.563
Genotype	AA	85 (29.6)	199 (30.8)	1.00	
	AG	161 (56.1)	328 (50.8)	1.27 (0.87-1.85)	0.213
	GG	41 (14.3)	119 (18.4)	1.08 (0.65-1.80)	0.771
Dominant	GG	41 (14.3)	119 (18.4)	1.00	
	AG + AA	246 (85.7)	527 (81.6)	1.08 (0.69-1.70)	0.744
Recessive	AA	85 (29.6)	199 (30.8)	1.00	
	AG + GG	202 (70.4)	447 (69.2)	1.22 (0.85-1.76)	0.273

^a^Adjusted for age, sex, smoking, drinking, hypertension, diabetes and hyperlipidemia

^b^HWE, Hardy–Weinberg equilibrium

After adjustment for possible confounding factors (age, sex, smoking, drinking, hypertension, diabetes and hyperlipidemia), the frequencies of the GG, AG, and AA genotypes of pre-miR27a rs895819 were 10.1%, 35.5%, 54.4% respectively among the cases, and 5.7%, 38.9%, 55.4% respectively among the controls. Subjects carrying AG heterozygote (OR = 0.40, 95% CI = 0.21-0.76, *P*_*a*_ = 0.005) or AA homozygote (OR = 0.40, 95% CI = 0.22-0.75, *P*_*a*_ = 0.004) had a reduced susceptibility of MI in comparison with GG homozygote (Table 2). Similarly, a trend of the reduced MI risk was detected when the AG and AA genotypes were combined (OR = 0.40, 95% CI = 0.22-0.74, *P*_*a*_ = 0.003, Table 2). However, no significant association between pri-miR-26a-1 pri-miR-100, pri-miR-126 and pri-miR-218 polymorphisms and MI risk was observed under the allelic and established genetic models (Table 2).

### Stratified analysis of pre-miR-27a rs895819 polymorphism with the risk of MI

We further evaluated the genotypes and MI susceptibility stratified by age, gender and status of smoking and drinking. As shown in Table 3, except for drinking status (OR = 0.41), the reduced risk associated with AG + AA genotypes was more pronounced among younger (OR = 0.34, 95% CI = 0.14-0.88, *P*a = 0.025), male (OR = 0.28, 95% CI = 0.13-0.61, *P*a = 0.001) and smoking (OR = 0.28, 95% CI = 0.10-0.78, *P*a = 0.015) subjects.

**Table 3:** Multivariate associations of the pre-miR-27a rs895819 with the risk of MI by further stratification for age, gender, smoking and drinking

Variables	Cases/controls	Genotype (cases/controls)		OR (95% CI) ^a^	*P* ^a^
		GG	AG + AA		
		No. (%)	No. (%)		
*Age*					
≤ 62	152/341	16/14 (10.5/4.1)	136/327 (89.5/95.9)	0.34 (0.14-0.88)	0.025
*Gender*					
Male	222/374	22/22 (9.9/5.9)	200/352 (90.1/94.1)	0.28 (0.13-0.61)	0.001
*Smoking*					
Yes	171/164	18/8 (10.5/4.9)	153/156 (89.5/95.1)	0.28 (0.10-0.78)	0.015
*Drinking*					
No	210/555	24/35 (11.4/6.3)	186/520 (88.6/93.7)	0.41 (0.22-0.77)	0.006

^a^Adjusted for age, gender, smoking and drinking within the strata

### Multivariate associations of the pre-miR-27a rs895819 polymorphism with the lipid profile

Since the pre-miR-27a rs895819 polymorphism may exert its effects on MI by regulating lipid metabolism, we further analyzed the association between rs895819 polymorphism and TG, TC, LDL-C and HDL-C levels. Our results found that AG and AA genotypes of the rs895819 polymorphism conferred about 0.17 mmol/L and 0.18 mmol/L increase in HDL-C levels compared to GG genotype (Table 4).

**Table 4:** ANOVA analysis of the association between pre-miR-27a rs895819 and the TG, TC, LDL-C and HDL-C levels

Variable	GG (*n* = 66)	AG (*n* = 353)	AA (*n* = 514)	*P*-value^a^
Triglycerides (mmol/L)	1.79 ± 1.30	1.64 ± 0.85	1.64 ± 0.86	0.422
Total cholesterol (mmol/L)	4.33 ± 1.27	4.65 ± 1.15	4.68 ± 1.16	0.072
LDL cholesterol (mmol/L)	2.70 ± 0.99	2.76 ± 0.90	2.77 ± 0.98	0.875
HDL cholesterol (mmol/L)	1.14 ± 0.31	1.31 ± 0.41	1.32 ± 0.61	**0.035** ^b^

^a^Two-sided chi-square test or independent-samples *t*-test

^b^*P* values under 0.05 were indicated in bold font

## Discussion

The present case-control study evaluated the potential association between five pri/pre-miRNA polymorphisms and MI susceptibility in a Chinese Han population. Our result showed that the pre-miR-27a rs895819 polymorphism was significantly associated with the MI risk, indicating that AG and AA genotypes in pre-miR-27a rs895819 polymorphism might have a protective effect against MI development compared with GG genotype.

Further stratification showed that the protective effect of AG + AA genotype was more remarkable among younger (≤ 62 years old), male and smoking subjects. The potential risk of MI in older subjects is more likely due to the aging effects as weak immune system and relative high-level exposure to environmental risk factors, rather than direct genetic effects. Sex-specific variation in atherosclerosis and CAD has been described [19, 20]. Hormonal differences, lifestyle, and other metabolism differences between males and females may elucidate our results.

MiR-27a has been reported not only to be an oncogenic miRNA [21], but also regulate lipid metabolism by altering the expression of many lipid metabolism-related genes [22]. It protected cardiomyocytes from mitochondria-mediated apoptosis during hypoxia/reperfusion injury by targeting IL-10-related pathways [23]. In addition, miR-27a played a unique role in the endothelial cells (ECs) dysfunction, which may contribute to the development of cardiovascular disease such as atherosclerosis, CAD and MI [24]. Urbich et al. has demonstrated that miR-27a promoted angiogenesis by targeting SEMA6A, which induces repulsion of neighboring endothelial cells [25]. Moreover, miR-27a has been shown to be associated with the renin-angiotensin system (RAS) and contribute to modulate the cardiovascular homeostasis and against aortic injury in hypertension by targeting ACE gene [26]. These studies indicate the multiple functions of miR-27a in different pathophysiological states that might be involved in the development and progression of cardiovascular diseases.

The pre-miR-27a rs895819 polymorphism has been reported to be significantly associated with the risk of type-2 diabetes (T2DM) [27] and a variety of cancers [28–30]. The original study can be traced from the time when Yang et al. firstly reported that the G-variant of rs895819 might impair the maturation of the miR-27a and thus, is associated with familial breast cancer risk [31]. Afterwards, GG genotype of this polymorphism was found significantly associated with an increased risk of T2DM in overweight subjects [27]. Here, we enlarge the knowledge of the effect of this polymorphism on human disease. In accordance with these studies, our results suggested that AG and AA genotype decreased the risk of MI, probably because these variants might affect the maturation of the miR-27a as described above, despite the marginal statistical significance of A allele compared with the G allele in reducing susceptibility of MI (Table 2). To further characterize the functional relevance of the miR-27a polymorphism, we conducted a correlation analysis between the genotypes and the expression of circulating mature miR-27a. However, our data shown that the relative expression of circulating miR-27a was not significantly different when compared the AG or AA genotype with the GG genotype (Additional file 2: Figure S1). Further study is required to clarify the underlying mechanism for this association.

Dysfunction of lipid metabolism is the fundamental pathogenesis of MI. MiR-27a inhibits the expression of many lipid metabolic genes, including FASN, SREBP-1, SREBP-2, PPARα and PPARγ, as well as ApoA1, ApoB100 and ApoE3A [22]. Additionally, a recent review of studies on miRNAs in lipid metabolism has determined that miR-27a may regulate lipid metabolism by reducing lipid synthesis and increasing lipid secretion from cells [32]. Our ANOVA analyses revealed that AG and AA genotypes increase the HDL-C in a significance level compared to GG genotype (Table 4). This result indicated that the mechanism contributing the decreased MI risk of pre-miR-27a rs895819 polymorphism might probably due to the elevated HDL-C levels.

Nevertheless, some limitations should be taken into consideration. First, we studied the association between rs895819 and MI risk in a Chinese Han population; further studies in different population are required to verify the true significance of their association. Second, the relatively moderate sample size limited the statistical power of our study. Third, the mechanisms by which the miR-27a and its targets regulate MI occurrence and progression are unknown, additional studies need to be performed prior to functional assessment of the link between rs895819 and MI.

## Conclusions

In conclusion, our findings firstly uncovered that the AG and AA genotypes in pre-miR-27a rs895819 polymorphism were associated with an decreased risk of MI in a Chinese Han population, and the association was more evident among younger, male and smoking subjects, which potentially due to the elevated HDL-C levels. However, further studies are warranted to confirm the general validity of these findings and to clarify the underlying mechanism for this association.

## Additional files

Additional file 1: Table S1. The sequences of the primers and probes used to genotype the rs895819 polymorphism. (DOCX 89 kb)

Additional file 2: Figure S1. Analysis of circulating mature miR-27a levels in three genotypes of 51 healthy controls. (DOCX 89 kb)

## Abbreviations

3’UTR: 3′ untranslated regions
CAD: Coronary artery disease
FBG: Fasting plasma glucose
HDL-C: High density lipoprotein cholesterol
LDL-C: Low density lipoprotein cholesterol
MI: Myocardial infarction
SNP: single nucleotide polymorphism
TC: Total cholesterol
TG: Triglyceride
