# Supplementary material for: The association between pre-miR-27a rs895819 polymorphism and myocardial infarction risk in a Chinese Han population
Source: Lipids Health Dis. 2018 Jan 6;17:7. doi: 10.1186/s12944-017-0652-x (PMC5756394; doi:10.1186/s12944-017-0652-x)
Supplement: Supplementary file 1 — The sequences of the primers and probes used to genotype the rs895819 polymorphism. (DOCX 89 kb) [file 12944_2017_652_MOESM1_ESM.docx]

Additional file 1

**Table S1. The sequences of the primers and probes used to genotype the polymorphisms.**

| **Name** | **Sequence (5’-3’)** |
| --- | --- |
| **Primers** |  |
| rs895819-F | ACTTAGCCACTGTGAACACG |
| rs895819-R | AGCAGGGCTTAGCTGCTTGT |
| rs7372209-F | AAAGGAGAGGCTGCCCAATG |
| rs7372209-R | CAGTCATGCTTACAGTCACG |
| rs1834306-F | GTCCCACTCTCACAAAAGC |
| rs1834306-R | GAAAAAGTGGAAACCAAGGG |
| rs4636297-F | ACCGTGAGTAATAATGCGCC |
| rs4636297-R | GAGGTCAAGGCTGAGGTCT |
| rs11134527-F | AGAAGCGGAAGCCGGAGCA |
| rs11134527-R | AATCTTCGGAAGTGTTCCAG |
| **Probes** |  |
| rs895819-FAM | P-CCAAGTCGTGTTCACAGTGGCTAAGTTTTTTTTTTTTTTTTTTTTTTT-FAM |
| rs895819-G | TTTTTTTTTTTTTTTTTTTTTTTTTTAGCTGCTTGTGAGCAGGGTCCACG |
| rs895819-A | TTTTTTTTTTTTTTTTTTTTTTTTTTTTAGCTGCTTGTGAGCAGGGTCCACA |
| rs7372209-FAM | P-AAGGATTAATTTCTCTCCTAATTCTTTTTTTTTTTTTTTTTTTTTTTTTTTTTTTTTTTTTTTTTTTTTTTTTTTTTTT-FAM |
| rs7372209-C | TTTTTTTTTTTTTTTTTTTTTTTTTTTTTTTTTTTTTTTTTTTTTTTTTTAGTCATGCTTACAGTCACGTGGTACG |
| rs7372209-T | TTTTTTTTTTTTTTTTTTTTTTTTTTTTTTTTTTTTTTTTTTTTTTTTTTTTAGTCATGCTTACAGTCACGTGGTACA |
| rs1834306-FAM | P-TGGGGAGAACATAGAAGATATTGCTTTTTTTTTTTTTTTTTTTTTTTTTTTTTTTTTTTTTTTTTTTTTTTTTTTTTTT-FAM |
| rs1834306-G | TTTTTTTTTTTTTTTTTTTTTTTTTTTTTTTTTTTTTTTTTTTTTTTTTTTTTTTTTTTTTTTTTTTTTTAAAAGTGGAAACCAAGGGAAGCACGC |
| rs1834306-A | TTTTTTTTTTTTTTTTTTTTTTTTTTTTTTTTTTTTTTTTTTTTTTTTTTTTTTTTTTTTTTTTTTTTTTTTAAAAGTGGAAACCAAGGGAAGCACGT |
| rs4636297-FAM | P-TTTCGATGCGGTGCCGTGGACGGCGTTTTTTTTTTTTTTTTTTTTTTTTTTTTTTTTTTTTTTTTTTTTTTTTTTTTTT-FAM |
| rs4636297-A | TTTTTTTTTTTTTTTTTTTTTTTTTTTTTTTTTTTTTTTTTTTTTTTTTTTTTTTTTTTTTTTTTGGTCAAGGCTGAGGTCTCAGCGGCGT |
| rs4636297-G | TTTTTTTTTTTTTTTTTTTTTTTTTTTTTTTTTTTTTTTTTTTTTTTTTTTTTTTTTTTTTTTTTTTGGTCAAGGCTGAGGTCTCAGCGGCGC |
| rs11134527-FAM | P-GCTCAGTGGGGGCCTGCTCCGGCTTTTTTTTTTTTTTTTTTTTTTTTTTTTTTTTTTTTTTTTTTTTTTTTTTTTTTTT-FAM |
| rs11134527-A | TTTTTTTTTTTTTTTTTTTTTTTTTTTTTTTTTTTTTTTTTTTTTTTTTTCCCACTCCTGATACTAATCAT |
| rs11134527-G | TTTTTTTTTTTTTTTTTTTTTTTTTTTTTTTTTTTTTTTTTTTTTTTTTTTTCCCACTCCTGATACTAATCAC |
