# Supplementary material for: The association between pre-miR-27a rs895819 polymorphism and myocardial infarction risk in a Chinese Han population
Source: Lipids Health Dis. 2018 Jan 6;17:7. doi: 10.1186/s12944-017-0652-x (PMC5756394; doi:10.1186/s12944-017-0652-x)
Supplement: Supplementary file 2 — Analysis of circulating mature miR-27a levels in three genotypes of 51 healthy controls. (DOCX 89 kb) [file 12944_2017_652_MOESM2_ESM.docx]

Additional file 2

**Fig. S1 Analysis of circulating mature miR-27a levels in three genotypes of 51 healthy controls.**
